# SARS-CoV-2 XEC: A Genome-Based Survey

**DOI:** 10.3390/microorganisms13020253

**Published:** 2025-01-24

**Authors:** Fabio Scarpa, Francesco Branda, Giancarlo Ceccarelli, Chiara Romano, Chiara Locci, Noemi Pascale, Ilenia Azzena, Pier Luigi Fiori, Marco Casu, Stefano Pascarella, Miriana Quaranta, Domenico Benvenuto, Roberto Cauda, Massimo Ciccozzi, Daria Sanna

**Affiliations:** 1 Department of Biomedical Sciences, University of Sassari, 07100 Sassari, Italy; c.locci3@phd.uniss.it (C.L.); fioripl@uniss.it (P.L.F.); darsanna@uniss.it (D.S.); 2 Unit of Medical Statistics and Molecular Epidemiology, Università Campus Bio-Medico di Roma, 00128 Rome, Italy; chiara.romano@unicampus.it (C.R.); m.ciccozzi@unicampus.it (M.C.); 3 Department of Public Health and Infectious Diseases, University of Rome Sapienza, 00161 Rome, Italy; giancarlo.ceccarelli@uniroma1.it; 4 Department of Veterinary Medicine, University of Sassari, Via Vienna 2, 07100 Sassari, Italy; npascale@uniss.it (N.P.); iazzena@uniss.it (I.A.); marcasu@uniss.it (M.C.); 5 Department of Chemical, Physical, Mathematical and Natural Sciences, University of Sassari, Via Vienna 2, 07100 Sassari, Italy; 6 Azienda Ospedaliera Universitaria (AOU) Sassari, 07100 Sassari, Italy; 7 Department of Biochemical Sciences “A. Rossi Fanelli”, Sapienza, 00185 Rome, Italy; stefano.pascarella@uniroma1.it (S.P.); miriana.quaranta@uniroma1.it (M.Q.); 8 Dipartimento di Sicurezza e Bioetica, Sezione di Malattie Infettive, Università Cattolica del Sacro Cuore, 00168 Rome, Italy; domenicobenvenuto95@gmail.com (D.B.); roberto.cauda@unicatt.it (R.C.)

**Keywords:** genetic diversity, SARS-CoV-2, XEC recombinant, viral recombination, genomic surveillance, phylogenomic analysis, spike protein mutations, evolutionary trajectory, pandemic monitoring

## Abstract

Recombination, a process of genetic exchange between distinct organisms, has played a critical role in the emergence of SARS-CoV-2 variants such as the XEC recombinant. This study provides a detailed genomic and structural characterization of XEC, derived from the recombination of lineages KP.3.3 (donor) and KS.1.1 (acceptor). Phylogenomic analyses reveal that XEC and its descendant XEC.1 form a monophyletic clade with close evolutionary ties to KP.3.3. The genomic breakpoint, spanning nucleotide positions 22,363–22,463, marks the shift from KS.1.1 to KP.3.3 within the spike protein gene. Mutational analysis highlights shared traits with its parental lineages, including mutations associated with immune evasion, receptor affinity, and fusogenicity. Notable changes, such as Q493E and L455S, may confer unique immunogenic properties, though XEC’s overall immune escape potential is limited by the absence of new mutations in conserved epitopes. Despite these mutations, XEC demonstrates restricted geographical spread, low genetic variability, and an evolutionary trajectory indicative of an evolutionary dead-end. Bayesian Skyline Plot analysis corroborates this, showing stable but declining population size. These findings underscore the need for ongoing genomic surveillance to monitor recombinant variants’ characteristics and public health impact. This study contributes to understanding viral evolution and highlights the importance of distinguishing variants of concern from those with minimal epidemiological significance.

## 1. Introduction

In recent years, the world has faced the COVID-19 pandemic, triggered by the SARS-CoV-2 virus, which was first identified in December 2019 during a pneumonia outbreak in Wuhan, China [1]. Initially confined to a few cases, the situation quickly escalated into a global crisis [2]. On 11 March 2020, with 149,295 confirmed cases worldwide, the World Health Organization (WHO) officially declared COVID-19 a pandemic [3]. SARS-CoV-2 is a single-stranded RNA virus with a positive-sense genome, characterized by a high error rate during RNA replication. This tendency to mutate influences its sensitivity to neutralizing antibodies generated through infection or vaccination, as well as its ability to spread [4]. As a result, throughout the pandemic, SARS-CoV-2 accumulated numerous mutations, giving rise to various lineages and sub-lineages.

Throughout the pandemic, SARS-CoV-2 experienced numerous mutations, leading to the emergence of multiple lineages and sub-lineages with varying capacities for spread [5]. Recombination events are also believed to significantly contribute to the development of new viral variants. In RNA viruses, recombination is typically a key mechanism of evolution [6], alongside re-assortment, which occurs specifically in RNA viruses with segmented genomes, such as the influenza virus [7]. For recombination between different lineages to occur, simultaneous infection by multiple viruses within the same host is required [8]. Continuous surveillance is essential to detect and monitor the emergence of new recombinants among recently identified variants [9,10].

The most recent recombinant lineage identified from a recombination event is SARS-CoV-2 XEC. This lineage is a hybrid of KP.3.3 and KS.1.1, both of which are descendants of the JN.1 lineage, which previously raised significant concerns [11]. XEC harbors five additional mutations in the spike protein compared to JN.1: T22N, F59S, F456L, Q493E, and V1104L.

A key concern, shared with its ancestor JN.1, is the presence of four mutations of interest (K417N, S477N, N501Y, and P681R) along with one mutation of concern (E484K). Although the precise role of these mutations is not yet fully understood, they warrant careful consideration. Notably, XEC is among the six Variants Under Monitoring (VUMs) tracked by the WHO (https://www.who.int/activities/tracking-SARS-CoV-2-variants, accessed on 6 December 2024) and was designated as a VUM on 24 September 2024 [12,13]. As with all variants, it is essential to maintain ongoing genomic surveillance, along with immunological and clinical monitoring, to detect and evaluate any mutations that might affect virus transmissibility, immune response, or pathogenicity. In this context, we conducted a genomic survey incorporating genetic variability, phylodynamic, and structural analyses to thoroughly assess the evolutionary potential, epidemiological behavior, and associated risks of XEC and its descendants. Specifically, XEC was analyzed to identify novel biological features that may contribute to its rapid spread or competitive advantage over the original lineages.

## 2. Materials and Methods

To place the SARS-CoV-2 XEC lineage in an evolutionary context, a preliminary phylogenomic analysis of Omicron variants was conducted. This analysis utilized global data via the nextstrain/ncov tool (https://github.com/nextstrain/ncov, accessed on 6 December 2024) and included all genomes from the GISAID isolated form from May 2024 to December 2024. A total of 1059 genomes out of 4009 were analyzed to understand the evolutionary trajectory. Genomes were filtered following GISAID’s criteria and selected based on sequence quality (excluding those with high ambiguity or gaps), balanced temporal and geographic representation, reduction in redundancy through strategic subsampling, prioritization of variants and lineages of interest, and the completeness of associated metadata. Following the initial phylogenomic assessment, genetic comparisons between XEC and its parental lineages were performed using three subsets: KP.3.3 (n = 246), KS.1.1 (n = 1006), and XEC (n = 184). The three subsets were constructed by selecting all available genomes for the three lineages under consideration, applying the filters “*Complete Genome*”, “*High Coverage*”, and “*Complete Collection Date*”. Independent genetic analyses were conducted on each subset, with details available in File S1. Genomes were aligned using the L-INS-I algorithm in MAFFT 7.471 [14]. Manual cleaning was made using Unipro UGENE v.35 [15], resulting in datasets of 29,715 (KP.3.3), 29,709 (KS.1.1), and 29,748 (XEC) base pairs. To determine the most suitable probabilistic model for genome evolution, the jModelTest 2.1.1 software [16] was used, employing a maximum likelihood optimized search. Bayesian Inference (BI) with BEAST 1.10.4 [17] was applied to estimate the times of the most recent common ancestor and evolutionary rates. This analysis included 400 million generations under various demographic and clock models, with the best-fit model selected via Bayes Factor tests [18] based on marginal likelihood comparisons, as outlined by Mugosa et al. [7]. Row files for Bayesian Skyline Plots (BSPs) and Lineages Through Time analyses were generated for SARS-CoV-2 XEC using BEAST, employing 400 million generations under the Bayesian Skyline Model with an uncorrelated log-normal relaxed clock. Graphs have been drawn by using the software Tracer 1.7.2 [19]. The software Tracer was also used to verify chain convergence and Effective Sample Size (ESS) values for all BEAST runs. The ESS values were always higher than 200 for all parameters.

For the parental lineages, this analysis involved only the estimation of the evolutionary rate. All genomes of each lineage were included, provided they met high-quality standards, had complete coverage, and included precise sampling dates (details in File S1). Genomic data for this study were sourced from the GISAID repository (https://gisaid.org/, accessed on 6 December 2024), and further information on the datasets and authorship is available in File S1. To investigate recombination events, a combined dataset of genomes from both parental lineages and the recombinant lineage (KP.3.3 + KS.1.1 + XEC) was used. Model-averaged support for breakpoint placement estimated using the algorithm GARD (Genetic Algorithm for Recombination Detection) [20] implemented in Datamonkey [21]. GARD is a tool designed to identify recombination events within a multiple sequence alignment. Recombination can complicate selection inference since recombinant sequences cannot be accurately represented by a single phylogenetic history, often resulting in a higher rate of false positives. By systematically analyzing alignments to locate recombination breakpoints, GARD addresses this issue, generating a distinct phylogenetic history for each identified recombination segment. Mutations defining these SARS-CoV-2 lineages were identified through consensus sequences, applying a 75% prevalence cutoff among all available sequences. This threshold aligns with the standards of the GISAID lineage comparison tool (https://gisaid.org/lineage-comparison/, accessed on 6 December 2024). The identified mutations were validated by cross-referencing results with the GISAID “Lineage Comparison” webpage (https://gisaid.org/lineage-comparison/, accessed on 6 December 2024).

Three-dimensional structures of the SARS-CoV-2 reference strain, KP.3.3 variant, XEC variant, and KS.1.1 variant were obtained using as a reference template the structure of the RBD-up state of the SARS-CoV-2 BA.2 variant spike glycoprotein (PDB: 8D56) [22]. Subsequently, DiscoTope 2.0 has been used to predict discontinuous B cell epitopes using the default DiscoTope score Threshold of −3.700 [23].

Homology models of the RBDs (receptor-binding domain) for JN.1, KP.3.3, KS.1.1, and XEC and their complexes with ACE2 were generated using Modeller 10.4 [24]. The structure identified by PDB code 6m0j was used as a template. Models were examined by using the graphical software PyMOL [25]. All structures were subjected to molecular dynamics simulations conducted using GROMACS 2024 [26], with the AMBER99SB-ILDN force field [27]. The structures were solvated in a dodecahedral box containing TIP3P water molecules, maintaining a 1.5 nm distance to the box edges. The system was neutralized and adjusted to a final concentration of 0.15 M NaCl. All simulations were performed under periodic boundary conditions. After energy minimization, the system underwent 100 ps of NVT and NPT equilibration at 300 K. Production simulations were carried out for 300 ns for the RBDs and 100 ns for the RBDs-ACE2 complexes, using a 2 fs time-step. Trajectories were visualized with VMD 1.9.3, and analysis was performed using GROMACS tools and the XMGRACE software package (Version 5.1.19) [28]. The net charge of the domains at pH 7.0 was calculated using the PROPKA3 software [29] on each of the 100 frames extracted from the entire simulation (one frame every 3 ns). The final net charge of each domain was the average charge calculated over the extracted frames. The standard error has been estimated from the distribution of the net charges within the frame set. The interaction energies between RBDs and ACE2 of all complexes were calculated using gmx_MMPBSA program [30] and a sampling interval of the molecular dynamic trajectories of 1 ns. The Poisson–Boltzmann solvation model was used. Specific interface interactions between RBDs and ACE2 were analyzed using per-residue free energy decomposition. To further validate the results, the trajectories from the dynamic simulations of the complexes were sampled every 10 ns, generating a total of 10 frames. These frames were then converted into structural (pdb) files and analyzed with DIMPLOT [31] to identify the interface interactions.

## 3. Results

### Phylodynamic

**Figure 1:**
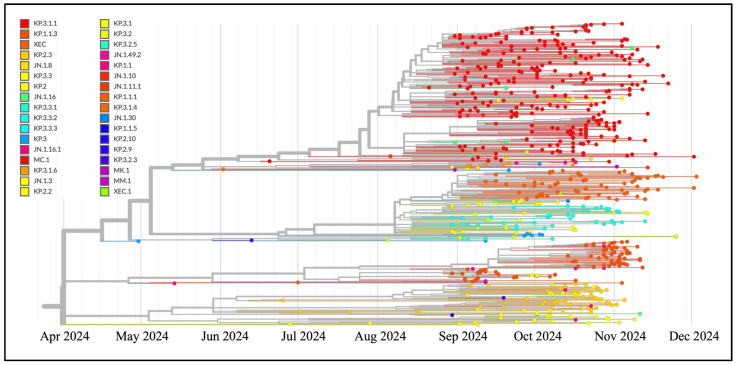
The time-scaled phylogenetic tree highlights strains within a global representative subsample of 491 out of 4063 SARS-CoV-2 genomes collected between April and December 2024. The phylogenetic analysis was performed using the nextstrain/ncov tool (https://github.com/nextstrain/ncov, accessed on 4 December 2024) and is available at https://gisaid.org/phylodynamics/global/nextstrain/ (accessed on 4 December 2024). The figure was refined using GIMP 2.8 software (available at https://www.gimp.org/downloads/oldstable/, accessed on 6 December 2024).

The phylogenomic analysis (Figure 1) reveals that XEC genomes cluster within an heterogenous clade that also include genomes of XEC, HN.1.8, KP.3.3, JN.1.16, KP.3.3.1, KP.3.3.2, KP.3.3.3, KP.3, JN.1.30, and XEC.1. The recombinant XEC and its first descendant XEC.1 clustered together in a monophyletic clade. The results of the Bayes Factor analysis across the three datasets indicate that the Bayesian Skyline Model, implemented under the lognormal uncorrelated relaxed clock model, provides a significantly better fit to the data compared to other tested demographic and clock models.

**Figure 2:**
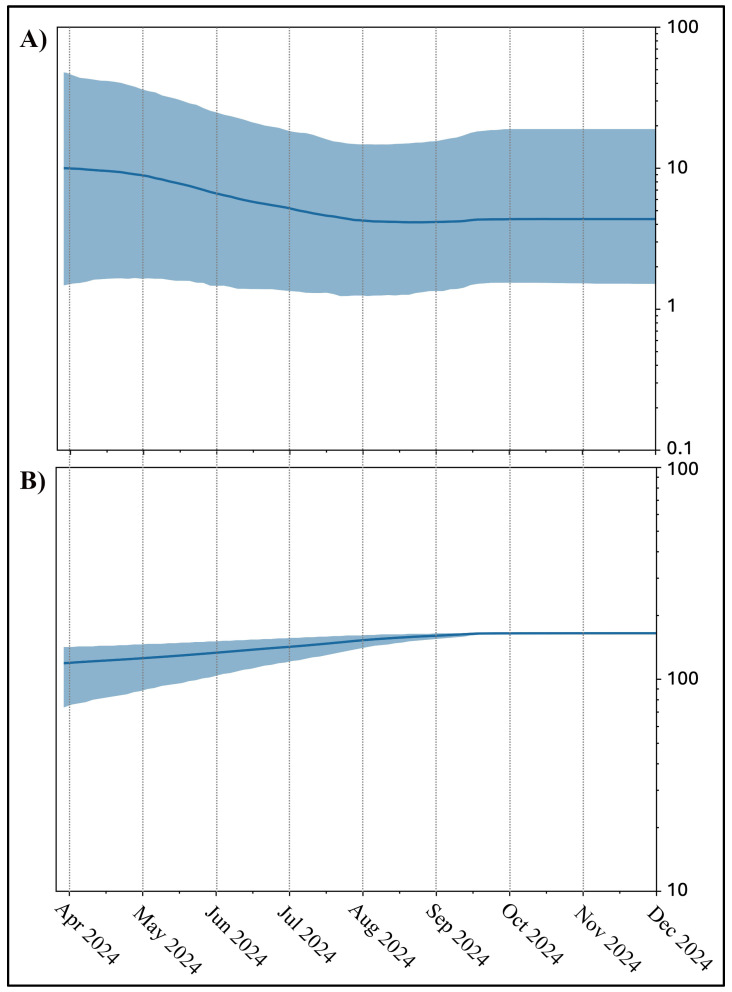
Bayesian Skyline Plot—BSP (**A**)—and Lineages Through Time—LLT (**B**)—of the recombinant lineage SARS-CoV-2 XEC. The viral effective population size (**A**) and the number of lineages (**B**) in the *y*-axis are plotted on the *y*-axis against the corresponding dates on the *x*-axis. The figure was refined using GIMP 2.8 software (available at https://www.gimp.org/downloads/oldstable/, accessed on 6 December 2024).

The recombination test indicated that the breakpoint is within the spike region, specifically between nucleotide positions 22,363 and 22.463 in the SARS-CoV-2 reference genome NC_045512.2 (800 and 900 in the spike region). The Bayesian Skyline Plot (BSP) of the recombinant XEC (Figure 2A) revealed a flattened genetic variability with slightly higher levels of genetic variability at the beginning, followed by a long final plateau, which persists to date. The Lineages Through Time (LTT) plot (Figure 2B) shows the lack of a growth in the number of lineages. The evolutionary rate of the three tested lineages amounts to 8.8 × 10^−4^ subs/sites/years (with a 95%HPD ranging between 5.37 × 10^−5^ and 1.66 × 10^−3^) for SARS-CoV-2 XEC and to 9.6 × 10^−5^ subs/sites/years (with a 95%HPD ranging between 2.15 × 10^−5^ and 1.86 × 10^−4^), 3.2 × 10^−5^ (with a 95%HPD ranging between 1.10 × 10^−5^ and 3.74 × 10^−4^) for the two parental lineages, KS.1.1 and KP.3.3, respectively.

The immunoinformatic analysis has found the presence of 97 B-cells epitope residues in the SARS-CoV-2 reference strain, 126 B-cells epitope residues in the XEC strain, 127 B-cells epitope residues in the KS.1.1 strain, and 124 B-cells epitope residues in the KP.3.3 strain. The main differences in terms of B-cells epitopes have been reported in Supplementary Material (Table S1).

The net charge of the different RBDs was calculated with PROPKA3 as a quantitative measure of the differences among the electrostatic surfaces during 300 ns of molecular dynamics simulations (Table 1). Compared to the JN.1 RBD, the net charge of the RBDs of the three variants KP.3.3, KS.1.1, and XEC decreased by approximately 13%. In the KP.3.3 and XEC variants, the appearance of the Q493E mutation, which replaces a neutral amino acid with a negatively charged one, contributed to this charge decrease. Similarly, the KS.1.1 R346T mutation replaces a positively charged amino acid with an uncharged one.

**Figure 3:**
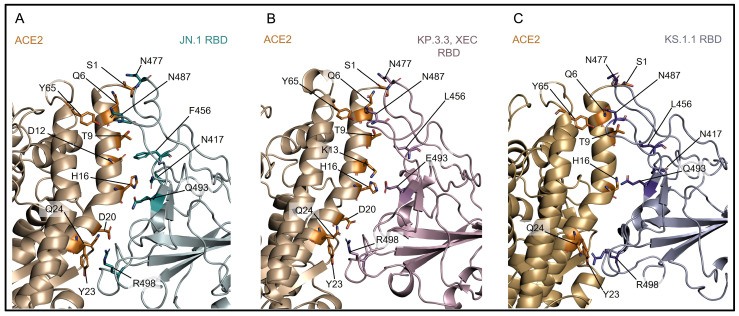
Comparison of the interfaces between ACE2 (orange) and the RBD of the JN.1 (**A**), KP.3.3 and XEC (**B**), and KS.1.1 (**C**) variants. Protein backbone is displayed as cartoon models while relevant side chains are depicted as labeled stick models.

The effect of the mutation at the RBD-ACE2 interface was evaluated by predicting the interaction energy of the different variants using the gmx_MMPBSA method (Table 2). The interaction of the RBD with ACE2 is more stable in the JN.1 variant compared to the three variants examined in this study. The residues primarily involved in the RBD-ACE2 interaction in the different variants are shown in Figure 3 and Table 3. The residue N417, which replaces K417 in all four variants, appears to interact with H16 of ACE2 in JN.1 and KS.1.1. However, in the KP.3.3 and XEC variants, this interaction does not occur. The increased distance between N417 and H16 in the KP.3.3 and XEC variants (Figure S1) can be caused by the presence of the Q493E mutation, which potentially induces local conformational changes. The impact of the Q493E mutation is shown in Figure 1 and Figure S2 and Table 3. Specifically, the Q493 residue (in JN.1 and KS.1.1) primarily interacts with H16 of ACE2, while E493 (in KP.3.3 and XEC) additionally forms an interaction with K13.

## 4. Discussion

Recombination is the process by which genetic material is exchanged between two distinct organisms, producing an “offspring” with a novel combination of traits absent in either parent [32]. The SARS-CoV-2 XEC recombinant emerged as a result of such an event during the ongoing COVID-19 pandemic. As with all newly identified variants, it is essential to thoroughly analyze XEC features, along with the differences from its parental lineages to assess its potential for transmission, immune evasion, and pathogenicity. This study used a genome-based approach to explore the evolutionary and structural characteristics of the SARS-CoV-2 recombinant XEC, utilizing all available GISAID genomes as of December 4, 2024. Phylogenomic reconstruction revealed that XEC genomes cluster within other evolutionary close lineages (XEC, HN.1.8, KP.3.3, JN.1.16, KP.3.3.1, KP.3.3.2, KP.3.3.3, KP.3, JN.1.30, and XEC.1), forming an heterogenous group, with XEC and its first descendant XEC.1 clustered together in a monophyletic clade. This placement indicates a close affinity in terms of mutations with the parental lineage KP.3.3, which in the recombination event acted as the donor lineage, while KS.1.1 served as the acceptor, following terminology by Focosi and Maggi [8]. Due to the close evolutionary relationship between the two parental variants, this region is highly conserved, containing shared mutations in both the parental variants and the recombinant. Consequently, it was not possible to determine the exact recombination point with greater precision. The initial portion of the XEC genome originates from KS.1.1, which functions as the acceptor in the recombination event, while KP.3.3 acts as the donor. Indeed, XEC shares a highly similar spike mutation profile with its parental lineages, differing by only two mutations from KP.3.3 (T22N and F59S) and four from KS.1.1 (R346T, Q493E, A1087S, and V1104L). Indeed, the behavior of their RBD domains is also very similar in the molecular dynamic simulation experiments. The recombination breakpoint, located between nucleotide positions 22,363 and 22,463 of the SARS-CoV-2 reference genome (NC_045512.2), spans amino acid positions 267–300 within the spike protein gene. This breakpoint aligns with the transition where XEC genomic composition shifts from KS.1.1 to KP.3.3. However, due to the region conserved nature, pinpointing the exact recombination site is challenging. The time-calibrated analysis suggests XEC likely originated in June 2024, approximately 2 months before the date of the earliest documented sample (i.e., 26 June 2024) [12]. During its early stages, the parental lineage KP.3.3 were prevalent in western Europe, while KS.1.1 were prevalent in Asia. Despite low prevalence levels, these parental lineages likely facilitated recombination. Initial expansion occurred in western Europe, where XEC reached a moderate genome lineage prevalence (https://gisaid.org/, accessed on 6 December 2024).

As also confirmed by its limited geographical spread, XEC shows limited evolutionary potential. Its phylogenomic pattern indicates an evolutionary dead-end, lacking significant diversification or epidemiological impact. This characteristic aligns with other variants, such as among others BA.2.75, BQ.1, and XBB, XBF, XBB.1.5, BA.2.86, and JN.1 which initially raised concerns but ultimately exhibited limited expansion capabilities [9,10,11,33,34]. Similar trends were observed with aal these variants which eventually disappeared after declining global prevalence. These observations are in line with the observed weakening of the interaction of KP.3.3, KS.1.1, and XEC RBD variants with ACE2 (Table 1). Bayesian Skyline Plot (BSP) analysis of XEC indicates very low levels of genetic variability and a small population size, with very mild changes over time. The graph indicates the lack of a real peak and depicts a linear level of genetic variability that after a constant level amounted to almost three months of decreases reaching the current level without further oscillations.

Overall, XEC, KS.1.1, and KP.3.3 variants have accumulated 17 mutations previously reported as responsible for increasing the immune evasion capability (G142D, del144/144, F157S, N211I, H245N, A264D, K356T, K417N, V445H, N450D, L452W, F456L, N460K, del483/483, E484K, F486P, and E554K). Six mutations previously reported as responsible for increasing the S-mediated fusogenicity (S50L, del212/212, H245N, A264D, D405N, and E484K), six mutations previously reported as responsible for increasing the affinity with ACE2 (V445H, L452W, N460K, N481K, E484K, and F456L), and five previously reported as responsible for increasing the cleavage efficacy in the cells (F157S, A264D, N460K, H655Y, and P681R) [35].

In particular, mutations N460K and F486P, also shared by XBB.1.5 and EG.5.1, are responsible for resistance to some RBD class 1 and, together with A484K and V483del, are responsible for resistance to some RBD class 2 mAbs [36]. K356T, L452W, and P445H are responsible for resistance to most antibodies in class 3 defined by their targeting epitope on RBD and contributed to the evasion of neutralizing antibodies in class 2 [37]. Though, XEC, KS.1.1, and KP.3.3 variants have three mutations (S50L, I332V, and R403K) that confer a degree of sensitization to neutralization by certain mAbs, it is important to highlight that no new mutation was found on the highly conserved 980–1006 conformational epitope that binds the 3A3 antibody [38]. In particular, it should be noted that KP.3.3 and XEC variants have a new aminoacidic change on the 493rd position (Q493E) that confers a more negative net charge compared to the previous variants. As previously reported, this position confers a higher sensitivity to certain mAbs and we can hypothesize that the Q493E may confer an even higher sensitivity to these mAbs. Regarding sensitivity to the anti-spike monoclonal antibodies, the F456L and L455S mutations (also known as Flip mutations) was found to be insensitive to bebtelovimab, Evusheld, and Sotrovimab (S-309) while preserving sensitivity to SA55, BD56-1854, S3H3, and Omi42. XEC, KS.1.1, and KP.3.3 variants have the F456L mutations but have a new mutation on the 455th position (L455S). Based on our calculation this mutation may probably have a more immunogenic potential, and it would be useful to ascertain if the available monoclonal antibodies preserve some activity against this variant [37,38]. By combining the results of our analysis with the literature data, it seems increasingly evident that the virus is sacrificing its affinity for the ACE2 receptor in favor of greater immunoevasive capability by decreasing its severity in favor of increased diffusivity through a process of gradual endemization.

Overall, the data presented here do not seem to present a worrying situation, but it is crucial to maintain a continuous surveillance with a multidisciplinary approach, providing insights that extend far beyond the characterization of a single variant.

Indeed, the genetic analysis of viruses plays a crucial role in understanding the mechanisms driving viral evolution, spread, and adaptation. By elucidating the molecular changes within the viral genome, researchers can identify patterns of mutation, recombination events, and their consequences on viral behavior, including transmissibility, immune evasion, and pathogenicity. In the context of a pandemic, genomic studies serve as an early warning system, allowing scientists to detect and monitor emerging variants with potential epidemiological or clinical significance. For instance, the ability to pinpoint recombination events, such as those that gave rise to XEC, enables us to understand how genetic material exchange between distinct lineages can produce recombinant viruses with novel traits. These studies not only help trace the evolutionary origins of variants but also provide critical information on how the virus may adapt to selective pressures such as host immunity, antiviral drugs, or monoclonal antibody treatments. The genomic data generated from such studies can also inform public health strategies. By analyzing genetic variability and evolutionary trajectories, researchers can assess the likelihood of a variant becoming widespread or posing a significant threat. In the case of XEC, its limited genetic variability and apparent evolutionary dead-end highlight that not all recombinants have the potential to cause global waves of infection. This underscores the importance of distinguishing between variants of concern and those with limited epidemiological impact. Moreover, genetic studies shed light on the mechanisms underlying immune evasion and resistance to therapeutic interventions. The identification of specific mutations, such as those affecting the receptor-binding domain (RBD) of the spike protein, helps predict how variants may respond to neutralizing antibodies or vaccines. For instance, mutations in XEC that influence antibody sensitivity provide a framework for assessing the efficacy of current monoclonal antibody therapies and adapting them to maintain their effectiveness. Beyond immediate public health responses, the integration of genetic insights into epidemiological models allows for more accurate forecasting of viral dynamics. For example, understanding how recombination hotspots or conserved genomic regions influence variant fitness can refine predictions about future evolutionary trends. This information is critical for vaccine development as it aids in identifying conserved targets less likely to mutate, ensuring longer-lasting immunity. The broader implications of such studies extend to global health policy and preparedness. Genomic monitoring can guide decisions on travel restrictions, resource allocation, and vaccination strategies by identifying regions where specific variants are emerging or spreading. It also emphasizes the need for international collaboration and data sharing, as demonstrated by platforms like GISAID, which provide the genomic data necessary for tracking and analyzing variants in near real-time.

## 5. Conclusions

In conclusion, XEC originated from a recombination event between the KP.3.3 (donor) and KS.1.1 (acceptor) lineages, with its genome demonstrating high similarity to its parental variants. Despite initial concerns, XEC shows limited evolutionary potential, as evidenced by its restricted geographical distribution, low genetic variability, and small population size. Its phylogenomic trajectory suggests an evolutionary dead-end, like other variants such as XBB and BA.2.86, which failed to sustain significant global prevalence. Our mutational analysis revealed that XEC, along with KS.1.1 and KP.3.3, carries mutations associated with immune evasion, enhanced ACE2 receptor affinity, and increased spike protein fusogenicity. Notably, while mutations like Q493E and L455S may confer unique immunogenic properties, the absence of new mutations in conserved epitopes limits its overall immune escape potential. This balance between immune evasion and receptor binding affinity reflects the broader trend of SARS-CoV-2 evolving towards decreased severity and increased transmissibility as part of its endemization process. These findings underscore the importance of continued genomic monitoring to track the emergence and characteristics of recombinant variants.

Indeed, these results must not be understood as a reason to let down the guard and every single new variant should be deeply analyzed. Future studies should further investigate the immunogenic implications of specific mutations and their impact on the efficacy of monoclonal antibody therapies.

## Figures and Tables

**Table 1:** RBD net charge of the SARS-CoV-2 lineage JN.1, KP.3.3, KS.1.1, and XEC.

	JN.1	KP.3.3	KS.1.1	XEC
PROPKA3	7.55 ± 0.01	6.53 ± 0.01	6.57 ± 0.01	6.53 ± 0.01

**Table 2:** RBD-ACE2 interaction energy expressed in kcal/mol of the SARS-CoV-2 lineage JN.1, KP.3.3, KS.1.1, and XEC.

	JN.1	KP.3.3	KS.1.1	XEC
MM/PBSA	−75.36 ± 0.71	−63.58 ± 1.86	−62.99 ± 0.99	−63.58 ± 1.86

**Table 3:** Energy contribution of the interface key residues expressed in kcal/mol.

Residue	int	vdw	eel	pol	tot
N417	25.04 ± 0.31	−5.62 ± 0.11	−89.28 ± 0.25	−5.03 ± 0.16	−74.89 ± 0.31
F456 (JN.1)	22.31 ± 0.31	−6.64 ± 0.11	0.54 ± 0.14	−1.09 ± 0.08	15.12 ± 0.35
L456 (KP.3.3, KS.1.1, XEC)	20.49 ± 0.26	−5.08 ± 0.12	−21.44 ± 0.11	−1.11 ± 0.06	−7.15 ± 0.30
N477	22.54 ± 0.31	−1.14 ± 0.11	−78.45 ± 0.47	−12.61 ± 0.37	−69.66 ± 0.26
N487	21.83 ± 0.26	−4.89 ± 0.11	−90.95 ± 0.22	−4.96 ± 0.13	−78.97 ± 0.24
Q493 (JN.1, KS.1.1)	27.04 ± 0.27	−6.36 ± 0.15	−84.26 ± 0.47	−1.22 ± 0.34	−64.81 ± 0.27
E493 (KP.3.3, XEC)	25.04 ± 0.31	−5.62 ± 0.11	−89.28 ± 0.25	−5.03 ± 0.16	−74.89 ± 0.31
R498 (JN.1)	22.31 ± 0.31	−6.64 ± 0.11	0.54 ± 0.14	−1.09 ± 0.08	15.12 ± 0.35
R498 (KP.3.3, KS.1.1, XEC)	20.49 ± 0.26	−5.08 ± 0.12	−21.44 ± 0.11	−1.11 ± 0.06	−7.15 ± 0.30

## Data Availability

Genomes analyzed in the present study were taken from GSAID database and are available at https://gisaid.org/, accessed on 4 December 2024. See File S1 for details.

## Supplementary Materials

The following supporting information can be downloaded at https://www.mdpi.com/article/10.3390/microorganisms13020253/s1. File S1: Acknowledgement and reference of authors who deposited analyzed genomes in GISAID portal. Figure S1: Minimum distance between the side chains of the residues N417 (RBD) and H16 (ACE2) calculated over the entire molecular dynamics simulation for the structural complexes of the JN.1 (black), KS.1.1 (red), and KP.3.3 and XEC (green) variants. Figure S2: Bar plots of the decomposition of energy components (kcal/mol) of the residue in position 493. Int = Internal energy contributions; vdw = van der Waals energy contributions; eel = electrostatic energy contributions; pol = polar solvation free energy contributions; tot = total free energy contributions (sum of all). Table S1: Aminoacidic position within the SARS-CoV-2 spike glycoprotein with the relative amino acid changes and the DiscoTope Scores have been reported for SARS-CoV-2 reference, XEC variant, KS.1.1 variant, and KP.3.3 variant; the probable B cells epitope have been highlighted in green.
