# Supplementary material for: SARS-CoV-2 XEC: A Genome-Based Survey
Source: Microorganisms. 2025 Jan 24;13(2):253. doi: 10.3390/microorganisms13020253 (PMC11857677; doi:10.3390/microorganisms13020253)
Supplement: Supplementary file 1 [file microorganisms-13-00253-s001.zip › File_S1.pdf]

SUPPLEMENTAL TABLE

Data Availability

GISAID Identifier:	EPI_SET_241120kz
doi:	10.55876/gis8.241120kz

All genome sequences and associated metadata in this dataset are published in GISAID's EpiCoV database. To view the contributors of each individual sequence with details such as accession number, Virus name, Collection date, Originating Lab and Submitting Lab and the list of Authors, visit 10.55876/gis8.241120kz

Data Snapshot

- EPI_SET_241120kz is composed of 1,205 individual genome sequences.
- The collection dates range from 2024-04-10 to 2024-11-09;
- Data were collected in 47 countries and territories;
- All sequences in this dataset are compared relative to hCoV-19/Wuhan/WIV04/2019 (WIV04), the official reference sequence employed by GISAID (EPI_ISL_402124). Learn more at https://gisaid.org/WIV04.

SUPPLEMENTAL TABLE

Data Availability

GISAID Identifier:	EPI_SET_241120se
doi:	10.55876/gis8.241120se

All genome sequences and associated metadata in this dataset are published in GISAID's EpiCoV database. To view the contributors of each individual sequence with details such as accession number, Virus name, Collection date, Originating Lab and Submitting Lab and the list of Authors, visit 10.55876/gis8.241120se

Data Snapshot

- EPI_SET_241120se is composed of 9,152 individual genome sequences.
- The collection dates range from 2024-03-17 to 2024-11-13;
- Data were collected in 47 countries and territories;
- All sequences in this dataset are compared relative to hCoV-19/Wuhan/WIV04/2019 (WIV04), the official reference sequence employed by GISAID (EPI_ISL_402124). Learn more at https://gisaid.org/WIV04.

SUPPLEMENTAL TABLE

Data Availability

GISAID Identifier: EPI_SET_241120xt
doi: 10.55876/gis8.241120xt

All genome sequences and associated metadata in this dataset are published in GISAID's EpiCoV database. To view the contributors of each individual sequence with details such as accession number, Virus name, Collection date, Originating Lab and Submitting Lab and the list of Authors, visit 10.55876/gis8.241120xt

Data Snapshot

- EPI_SET_241120xt is composed of 13,315 individual genome sequences.
- The collection dates range from 2024-03-08 to 2024-11-12;
- Data were collected in 55 countries and territories;
- All sequences in this dataset are compared relative to hCoV-19/Wuhan/WIV04/2019 (WIV04), the official reference sequence employed by GISAID (EPI_ISL_402124). Learn more at https://gisaid.org/WIV04.
