# Supplementary material for: SARS-CoV-2 XEC: A Genome-Based Survey
Source: Microorganisms. 2025 Jan 24;13(2):253. doi: 10.3390/microorganisms13020253 (PMC11857677; doi:10.3390/microorganisms13020253)
Supplement: Supplementary file 1 [file microorganisms-13-00253-s001.zip › Table_S1.pdf]

Supplementary Table S1. Aminoacidic position within the SARS-Cov-2 Spike glycoprotein with the relative amino acid changes and the DiscoTope Scores have been reported for SARS-CoV-2 reference, XEC variant, KS.1.1 variant and KP.3.3 variant, the probable B cells epitope have been highlighted in green

Residue ID	Reference Residue Name	XEC Residue Name	KS.1.1 Residue Name	KP.3.3 Residue Name	Reference Discotope Score	XEC Discotope Score	KS.1.1 Discotope Score	KP.3.3 Discotope Score
73	**THR**	THR	THR	THR	**-2.295**	-8.109	-8.158	-8.642
74	**ASN**	ASN	ASN	ASN	**-0.797**	-5.03	-4.405	-5.53
145	**TYR**				**-3.184**			
146	**HIS**	**HIS**	**HIS**	**HIS**	**-1.434**	**-2.422**	**-2.422**	**-2.421**
147	**LYS**	**LYS**	**LYS**	**LYS**	**2.203**	**-1.174**	**-1.178**	**-1.178**
148	**ASN**	**ASN**	**ASN**	**ASN**	**3.046**	**2.554**	**2.553**	**2.553**
149	**ASN**	**ASN**	**ASN**	**ASN**	**1.945**	**3.23**	**3.229**	**3.229**
150	**LYS**	**LYS**	**LYS**	**LYS**	**3.317**	**3.352**	**3.573**	**3.352**
151	**SER**	**SER**	**SER**	**SER**	**0.722**	**1.245**	**1.247**	**1.244**
179	LEU	**LEU**	**LEU**	**LEU**	-4.22	**-2.829**	**-2.836**	**-2.832**
180	**GLU**	**GLU**	**GLU**	**GLU**	**1.494**	**1.999**	**1.993**	**1.997**
181	**GLY**	**GLY**	**GLY**	**GLY**	**2.746**	**3.308**	**3.303**	**3.307**
182	**LYS**	**LYS**	**LYS**	**LYS**	**1.021**	**2.408**	**2.405**	**2.407**
183	**GLN**	**GLN**	**GLN**	**GLN**	**0.831**	**2.342**	**2.338**	**2.341**
184	**GLY**	**GLY**	**GLY**	**GLY**	**1.447**	**3.164**	**3.162**	**3.165**
185	**ASN**	**ASN**	**ASN**	**ASN**	**-1.167**	**1.084**	**1.079**	**1.083**
186	**PHE**	**PHE**	**PHE**	**PHE**	**-2.584**	**-0.391**	**-0.406**	**-0.397**
209	PRO	**PRO**	**PRO**	**PRO**	-4.792	**-2.636**	**-2.646**	**-2.854**
211	**ASN**	**ILE**	**ILE**	**ILE**	**-1.84**	**0.51**	**0.503**	**0.301**
212	**LEU**				**-3.601**			
213	VAL	**GLY**	**GLY**	**GLY**	-3.861	**0.492**	**0.608**	**0.272**
214	ARG	**ARG**	**ARG**	**ARG**	-5.993	**-0.347**	**-0.354**	**-0.66**
215	ASP	**ASP**	**ASP**	ASP	-12.042	**-3.283**	**-3.491**	-4.245
250	**THR**	**THR**	**THR**	**THR**	**-0.689**	**-0.516**	**-0.516**	**-0.517**
251	**PRO**	**PRO**	**PRO**	**PRO**	**-1.744**	**1.488**	**1.61**	**1.61**
252	**GLY**	**GLY**	**GLY**	**GLY**	**0.285**	**0.557**	**0.559**	**0.558**
253	**ASP**	**ASP**	**ASP**	**ASP**	**0.515**	**-0.256**	**-0.257**	**-0.258**
254	**SER**	**SER**	**SER**	**SER**	**-2.329**	**-1.352**	**-1.349**	**-1.353**
255	**SER**	SER	SER	SER	**-2.169**	-6.427	-6.551	-6.431
415	**THR**	**THR**	**THR**	**THR**	**-3.544**	**-3.428**	**-3.276**	**-3.42**
417	LYS	**ASN**	**ASN**	ASN	-5.918	**-3.629**	**-3.528**	-3.83
440	**ASN**	**LYS**	**LYS**	**LYS**	**-3.205**	**-2.684**	**-2.992**	**-2.452**

443	**SER**	**SER**	**SER**	**SER**	**-3.184**	**-1.868**	**-2.348**	**-1.871**
444	**LYS**	**LYS**	**LYS**	**LYS**	**-0.428**	**1.361**	**0.987**	**1.359**
445	**VAL**	**HIS**	**HIS**	**HIS**	**2.915**	**4.841**	**4.607**	**4.841**
446	**GLY**	**SER**	**SER**	**SER**	**3.412**	**4.782**	**4.646**	**4.78**
447	**GLY**	**GLY**	**GLY**	**GLY**	**0.867**	**3.004**	**2.824**	**3.003**
448	**ASN**	**ASN**	**ASN**	**ASN**	**-3.145**	**-1.672**	**-1.983**	**-1.555**
449	**TYR**	**TYR**	**TYR**	**TYR**	**-1.851**	**0.819**	**0.661**	**0.82**
450	ASN	**ASP**	**ASP**	**ASP**	-4.024	**-1.735**	**-2.037**	**-1.736**
452	LEU	**TRP**	TRP	**TRP**	-6.623	**-3.685**	-3.794	**-3.686**
454	ARG	**ARG**	**ARG**	**ARG**	-7.238	**-3.597**	**-3.523**	**-3.595**
455	LEU	**SER**	**SER**	**SER**	-5.275	**-0.978**	**-0.581**	**-0.977**
456	PHE	**LEU**	**LEU**	**LEU**	-7.046	**-2.741**	**-2.374**	**-2.74**
457	ARG	**ARG**	**ARG**	**ARG**	-5.489	**-1.34**	**-1.053**	**-1.34**
458	**LYS**	**LYS**	**LYS**	**LYS**	**-2.91**	**0.976**	**1.181**	**0.977**
459	**SER**	**SER**	**SER**	**SER**	**-1.721**	**1.425**	**1.596**	**1.426**
460	**ASN**	**LYS**	**LYS**	**LYS**	**-2.72**	**-1.115**	**-0.942**	**-1.115**
462	**LYS**	**LYS**	**LYS**	**LYS**	**-2.902**	**-2.79**	**-2.789**	**-2.788**
468	ILE	**ILE**	**ILE**	**ILE**	-4.359	**-2.335**	**-2.429**	**-2.335**
469	SER	**SER**	**SER**	**SER**	-5.734	**-2.848**	**-2.557**	**-2.849**
470	THR	**THR**	**THR**	**THR**	-6.09	**-2.241**	**-2.151**	**-2.241**
471	GLU	**GLU**	**GLU**	**GLU**	-5.755	**-1.541**	**-1.215**	**-1.541**
472	ILE	**ILE**	**ILE**	**ILE**	-7.04	**-1.922**	**-1.585**	**-1.92**
473	TYR	**TYR**	**TYR**	**TYR**	-6.769	**-1.707**	**-1.397**	**-1.707**
474	GLN	**GLN**	**GLN**	**GLN**	-5.555	**-0.16**	**0.087**	**-0.159**
475	ALA	**ALA**	**ALA**	**ALA**	-7.216	**-1.922**	**-1.65**	**-1.92**
476	GLY	**GLY**	**GLY**	**GLY**	-5.974	**-0.721**	**-0.507**	**-0.721**
477	SER	**ASN**	**ASN**	**ASN**	-3.77	**1.459**	**1.61**	**1.459**
478	THR	**LYS**	**LYS**	**LYS**	-4.716	**0.114**	**0.036**	**-0.105**
479	PRO	**PRO**	**PRO**	**PRO**	-4.07	**0.353**	**0.509**	**0.354**
480	CYS	**CYS**	**CYS**	**CYS**	-7.312	**-1.905**	**-1.708**	**-1.904**
481	ASN	**LYS**	**LYS**	**LYS**	-4.865	**-0.159**	**0.07**	**-0.158**
482	GLY	**GLY**	**GLY**	**GLY**	-5.335	**-0.34**	**-0.028**	**-0.339**
484	GLU	**LYS**	**LYS**	**LYS**	-5.461	**-0.059**	**0.267**	**-0.058**
485	GLY	**GLY**	**GLY**	**GLY**	-7.501	**-1.757**	**-1.374**	**-1.643**
486	PHE	**PRO**	**PRO**	**PRO**	-5.666	**-0.053**	**0.186**	**-0.054**
487	ASN	**ASN**	**ASN**	**ASN**	-5.342	**0.055**	**0.354**	**0.055**
488	CYS	**CYS**	**CYS**	**CYS**	-8.077	**-2.447**	**-2.218**	**-2.56**
489	TYR	**TYR**	**TYR**	**TYR**	-7.01	**-2.09**	**-1.66**	**-2.088**
490	PHE	**PHE**	**PHE**	**PHE**	-7.209	**-2.729**	**-2.279**	**-2.612**

491	PRO	PRO	PRO	PRO	-5.724	-1.585	-1.454	-1.7
492	LEU	LEU	LEU	LEU	-5.345	-0.93	-0.731	-0.929
493	GLN	GLU	GLN	GLU	-3.084	1.004	1.228	1.005
494	SER	SER	SER	SER	-5.835	-1.976	-1.987	-1.975
495	TYR	TYR	TYR	TYR	-4.794	-2.857	-3.092	-2.856
496	GLY	GLY	GLY	GLY	-0.505	1.024	0.967	1.023
497	PHE	PHE	PHE	PHE	-4.121	-2.673	-2.912	-2.675
498	GLN	ARG	ARG	ARG	1.184	2.739	2.518	2.737
499	PRO	PRO	PRO	PRO	0.18	1.043	0.739	1.041
500	THR	THR	THR	THR	2.994	3.825	3.636	3.824
501	ASN	TYR	TYR	TYR	1.676	2.087	1.99	2.086
502	GLY	GLY	GLY	GLY	-1.969	-1.226	-1.371	-1.227
503	VAL	VAL	VAL	VAL	0.721	1.042	0.9	1.04
504	GLY	GLY	GLY	GLY	-2.554	-2.335	-2.503	-2.333
505	TYR	HIS	HIS	HIS	-1.749	-1.438	-1.6	-1.439
506	GLN	GLN	GLN	GLN	-4.384	-3.538	-3.827	-3.54
556	ASN	ASN	ASN	ASN	-2.977	-2.571	-2.566	-2.569
558	LYS	LYS	LYS	LYS	-1.939	-1.511	-1.506	-1.51
560	LEU	LEU	LEU	LEU	-3.266	-2.968	-2.968	-2.969
678	THR	THR	THR	THR	-2.983	-3.414	-3.429	-3.419
679	ASN	LYS	LYS	LYS	-0.547	-3.543	-3.559	-3.549
680	SER	SER	SER	SER	2.385	0.22	0.107	0.106
681	PRO	ARG	ARG	ARG	1.491	0.415	0.414	0.415
682	ARG	ARG	ARG	ARG	1.254	0.773	0.771	0.773
683	ARG	ARG	ARG	ARG	2.102	2.231	2.227	2.23
684	ALA	ALA	ALA	ALA	1.429	0.538	0.537	0.538
685	ARG	ARG	ARG	ARG	0.544	0.701	0.438	0.44
686	SER	SER	SER	SER	0.92	1.052	1.048	1.051
687	VAL	VAL	VAL	VAL	0.966	0.32	0.311	0.318
688	ALA	ALA	ALA	ALA	-0.579	-2.213	-2.214	-2.213
703	ASN	ASN	ASN	ASN	-2.45	-2.45	-2.45	-2.45
704	SER	SER	SER	SER	-1.82	-1.82	-1.821	-1.82
705	VAL	VAL	VAL	VAL	-3.581	-3.586	-3.581	-3.586
716	THR	THR	THR	THR	-3.642	-3.811	-3.643	-3.81
793	PRO	PRO	PRO	PRO	-1.392	-1.78	-1.779	-1.779
794	ILE	ILE	ILE	ILE	-2.217	-2.675	-2.673	-2.674
809	PRO	PRO	PRO	PRO	-2.112	-2.947	-2.95	-2.948
810	SER	SER	SER	SER	0.807	-0.128	-0.131	-0.129
811	LYS	LYS	LYS	LYS	-2.839	-4.528	-4.535	-4.53

812	PRO	PRO	PRO	PRO	-0.921	-2.94	-2.943	-2.94
914	ASN	ASN	ASN	ASN	-1.359	-1.631	-1.497	-1.63
917	TYR	TYR	TYR	TYR	-3.443	-3.761	-3.677	-3.76
918	GLU	GLU	GLU	GLU	-3.66	-3.943	-3.84	-3.941
1100	THR	THR	THR	THR	-3.546	-3.711	-3.489	-3.709
1118	ASP	ASP	ASP	ASP	-3.671	-5.21	-4.349	-5.209
1140	PRO	PRO	PRO	PRO	-1.938	-4.122	-3.596	-4.121
1141	LEU	LEU	LEU	LEU	-1.03	-3.212	-2.762	-3.211
1142	GLN	GLN	GLN	GLN	-0.755	-2.899	-2.456	-2.899
1143	PRO	LEU	LEU	LEU	0.104	-2.643	-2.565	-2.642
1144	GLU	GLU	GLU	GLU	0.834	-1.337	-1.341	-1.336
1145	LEU	LEU	LEU	LEU	0.291	-1.829	-1.831	-1.828
1146	ASP	ASP	ASP	ASP	1.077	-1.08	-1.084	-1.078
1147	SER	SER	SER	SER	0.431	-1.63	-1.626	-1.628
1148	PHE	PHE	PHE	PHE	0.407	-1.415	-1.415	-1.414
1149	LYS	LYS	LYS	LYS	0.812	-0.9	-0.902	-0.901
1150	GLU	GLU	GLU	GLU	1.524	-0.114	-0.113	-0.113
1151	GLU	GLU	GLU	GLU	1.088	-0.385	-0.384	-0.384
1152	LEU	LEU	LEU	LEU	0.895	-0.4	-0.4	-0.4
1153	ASP	ASP	ASP	ASP	1.33	0.136	0.137	0.137
1154	LYS	LYS	LYS	LYS	1.536	0.46	0.459	0.46
1155	TYR	TYR	TYR	TYR	1.142	0.252	0.248	0.251
1156	PHE	PHE	PHE	PHE	1.523	0.79	0.791	0.791
1157	LYS	LYS	LYS	LYS	1.658	1.011	1.011	1.011
1158	ASN	ASN	ASN	ASN	1.377	1.376	1.374	1.376
1159	HIS	HIS	HIS	HIS	1.254	1.254	1.254	1.254
1160	THR	THR	THR	THR	1.569	1.57	1.569	1.569
