# Supplementary figures and images for: SARS-CoV-2 XEC: A Genome-Based Survey
Source: Microorganisms. 2025 Jan 24;13(2):253. doi: 10.3390/microorganisms13020253 (PMC11857677; doi:10.3390/microorganisms13020253)

# Minimum Distance

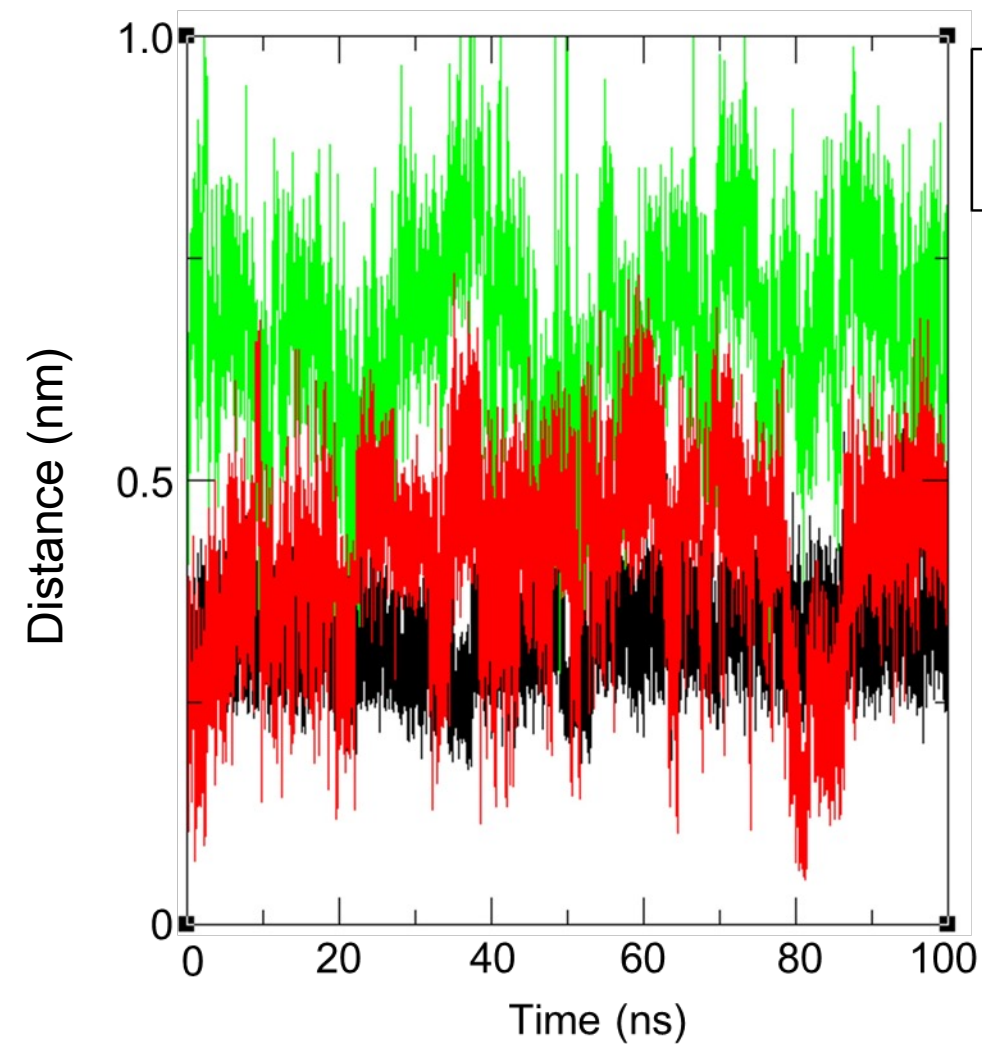

- N417 (JN.1) – H16 (ACE2)
- N417 (KS.1.1) – H16 (ACE2)
- N417 (KP.3.3, XEC) – H16 (ACE2)

Supplement: Supplementary file 1 [file microorganisms-13-00253-s001.zip › Figure_S1.pdf]

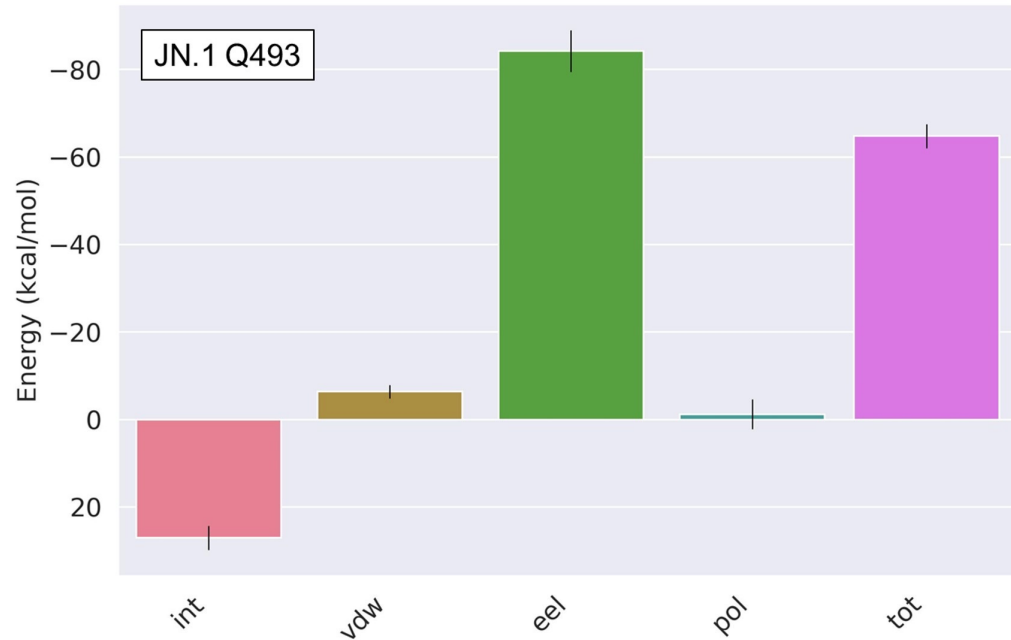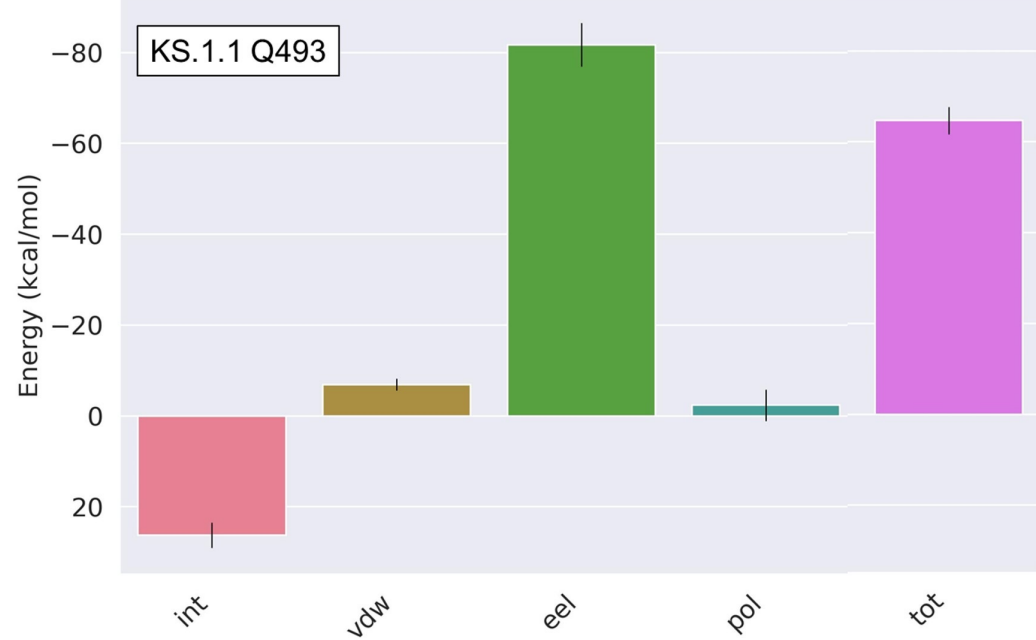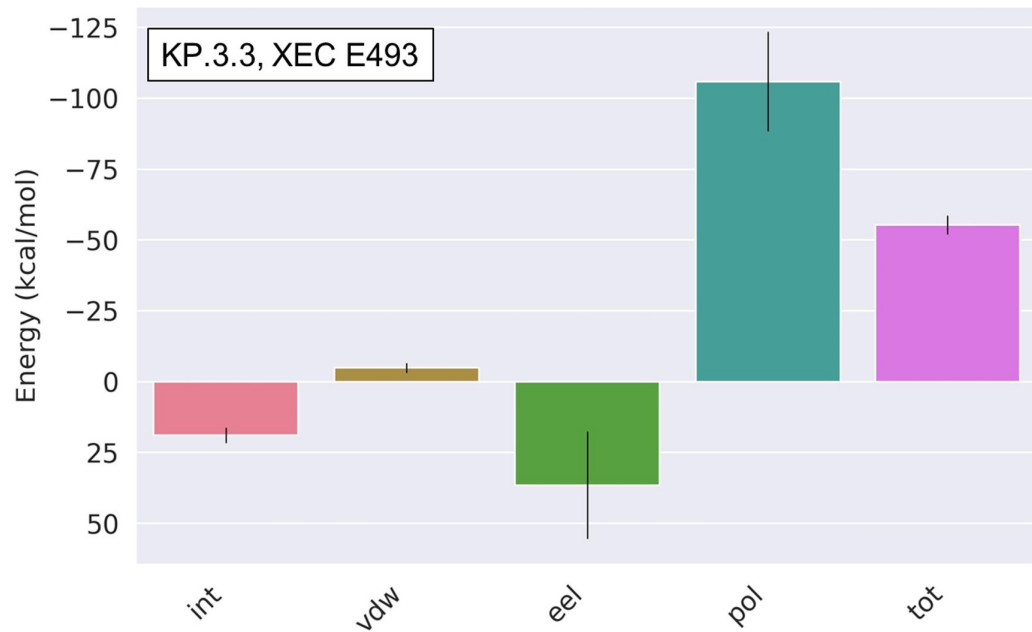

Supplement: Supplementary file 1 [file microorganisms-13-00253-s001.zip › Figure_S2.pdf]
